# Professional nurses’ experiences of caring for patients in public health clinics in Ekurhuleni, South Africa

**DOI:** 10.4102/phcfm.v11i1.1963

**Published:** 2019-06-10

**Authors:** Tintswalo V. Nesengani, Charlene Downing, Marie Poggenpoel, Chris Stein

**Affiliations:** 1 Department of Nursing, University of Johannesburg, Johannesburg, South Africa; 2 Department of Emergency Medicine, University of Johannesburg, Johannesburg, South Africa

**Keywords:** caring, professional nurses, empowering, qualitative research, disempowering

## Abstract

**Background:**

Caring for patients is the core aspect of nursing and a cornerstone of all nursing duties. Although caring is seen as a critical component of nursing delivery and an essential characteristic of nursing, there seems to be a gap between theory and practice.

**Aim:**

The aim of this article was to explore and describe the experiences of caring for patients by professional nurses in public health clinics in Ekurhuleni.

**Setting:**

The study was conducted in Ekurhuleni, an area east of the Gauteng Province in two public health clinics.

**Methods:**

A qualitative, exploratory, descriptive phenomenological and contextual research design was used. In-depth, individual phenomenological interviews were conducted with eight purposefully sampled professional nurses to explore their experiences of caring for patients in public health clinics in Ekurhuleni. Data were analysed using Giorgi’s coding method.

**Results:**

Two themes were revealed in the study findings. The first theme was the experienced empowering aspects of caring while the second theme was the experienced disempowering aspects of caring. The experienced empowering aspects of caring had two categories: empowering interpersonal experiences and the empowering experiences through client affirmation. These were identified by the participants as enabling effective caring for patients. The experienced disempowering aspects of caring also had two categories: disempowering interpersonal experiences and the disempowering experiences resulting from public health clinic system challenges. The disempowering aspects were identified by participants as disenabling effective caring for patients.

**Conclusion:**

The study findings reveal that the professional nurses had empowering and disempowering experiences while caring for patients in the public health clinics.

## Introduction

Caring is an act associated with assisting others, accompanied by compassion, kindness, empathy, respect, helpfulness, patience, mercy and integrity. While regarded as an essential feature and expression of being human, caring is widely accepted as a core characteristic of nursing.^1^ Caring actions by professional nurses are essentially related to helping patients to alleviate their pain and distress in a systematic way, while also being associated with the qualities of respect, patience, trust, honesty, communication, dedication and a positive attitude. As a moral ideal and an essential ingredient, nursing actions attempt to protect, enhance and preserve humanity. Caring must be present if nursing is to be truly effective and give patients a feeling of importance. A caring attitude is vital in the nursing profession to ensure the development of trust in the nurse–patient relationship.^2^

Nursing is a caring profession that requires the provision of excellent care within an ethical, reflective and knowing framework.^2^ Caring entails a helping attitude through guiding, advising and providing moral support by encouraging, listening and offering counselling skills. These skills are said to have enabled professional nurses to motivate patients to cope with various diseases by offering care that was patient-oriented and rooted in the needs of the individual patient.^3^

Although caring is generally viewed as the core of nursing actions that produce therapeutic results in the person being served – with the emphasis being placed on caring as crucial to nursing – there seems to be a gap between theory and practice. It then appears that theory and practice are two different things. Caring in nursing should help patients feel better, while the absence of caring will affect patients psychologically, emotionally and physically.^2^ Governments and nursing bodies have therefore indicated that patients have a right to be treated with care and compassion; however, reports show that not all patients receive compassionate caring from the nursing staff.^4^

According to a survey conducted in 2010, *Consolidated Report on Inspections of Primary Health Care Delivery Sites* on patients in various clinics in Gauteng, South Africa, it was revealed that nurses were lacking in caring.^4^ Patients revealed that nurses made rude, insulting and abusive remarks towards them. Other aspects revealed by patients included a lack of confidentiality with regard to information entrusted to professional nurses because, while at the clinics, they often overheard nurses gossiping about patients’ illnesses. In South Africa, clinics have become the cornerstone of the public health system. It is thus necessary and expected that clinics provide comprehensive and integrated basic health programmes, with members of the public being treated with caring, respect and compassion by all health professionals.^4^

The authors of this article have observed professional nurses to lack a helping attitude and the qualities of dedication in caring for patients. Some patients have been observed to face verbal abuse and substandard care, while others were turned away from the public health clinics without being examined. Such dismissive behaviour by professional nurses has also been reported in various public health clinics in the area concerned. This study’s findings identified empowering and disempowering aspects of caring as the main themes. Both themes have two categories each, with those for the empowering experiences identified as empowering interpersonal experiences and empowering experiences through client affirmation. The identified categories for the disempowering aspects of caring were disempowering interpersonal experiences and disempowerment resulting from public health clinic system challenges. Participants in this study identified the empowering aspects of caring as enabling them to render effective caring for patients while the aspect of feeling disempowered because of the public health clinic system challenges was regarded as disenabling. Caring experiences can therefore be empowered through encouraging the maintenance of the important enablers of effective caring for patients such as cooperation, teamwork, collaboration with other colleagues while focusing on the patients’ best interests, effective communication by the professional nurses and maintenance of coping strategies such as strong support systems. Caring experiences can be disempowered through factors such as long queues that await the professional nurses each morning, subjecting them to work under immense pressure on a daily basis. Other factors that can disempower caring experiences were mentioned by participants as shortage of nursing staff, shortage of functional medical equipment, limited budget allocation, uncaring behaviours displayed by some professional nurses, language barriers, especially while caring for foreign patients, shortage of medicines and the unavailability of ambulances. The need to address the disempowering experiences was evident in this study as participants indicated that being disempowered by all the factors identified led to deterioration in the quality of caring rendered to patients. The purpose of this study was therefore to gain insight into the professional nurses’ experiences of caring for patients.

## Research methods and design

### Study design

A qualitative, exploratory, descriptive phenomenological and contextual design was used. The fundamental point of departure for the researcher to use the qualitative research design was the fact that the qualitative approach is associated with naturalistic inquiry, which focuses on the way people make sense of their experiences and the world in which they live. The use of the exploratory research design was aimed at exploring the phenomenon of interest and shedding light on the various ways in which the phenomenon is expressed.^5^ The descriptive phenomenological research design was appropriate for this study as one of the research purposes was to describe professional nurses’ experiences of caring in the clinics. The rationale for the use of the descriptive phenomenological research design was that it involves careful description of ordinary conscious experience of everyday life, which is a description of ‘things’ as people experience them. Descriptive phenomenological research design includes such steps as bracketing, intuiting, analysing and describing.^6^ Bracketing assisted the researcher to identify and hold in abeyance preconceived beliefs and opinions about the phenomenon under study. Intuiting was used to help the researcher to remain open to the meanings attributed to the phenomenon by the participants who experienced it. Analysis enabled the researcher to extract significant statements, categorise and to make sense of the essential meanings of the phenomenon. Describing occurred when the researcher understood and defined the phenomenon. The contextual research design was chosen to assist the researchers to explore, interpret and describe the participants’ experiences in detail, in terms of their immediate environment or context.^5^

### Setting

This study was conducted in Ekurhuleni, a metropolitan municipality that forms the local government of the East Rand Region of the Gauteng Province, South Africa. South Africa is a country that comprises nine provinces, with Gauteng being the smallest province of the nine. The Gauteng Province encompasses about 3 379 104 people of diverse cultures, languages and belief practices.^7^ The name *Ekurhuleni* is derived from the Xitsonga language, in which it means ‘place of peace’. Ekurhuleni is one of the most densely populated areas in the province and the country; it has 94 public health clinics and seven public hospitals that render health care services to the community. The public health clinics provide primary health care services at a local level through the district health system, which is part of the provincial health care system.

### Population and sampling

The focus of this study was only on the public health clinics because professional nurses in these health care facilities were observed to be lacking in a helpful attitude and the qualities of dedication in caring for patients. Purposive sampling was used to select 2 of the 94 public health clinics in Ekurhuleni. The two public health clinics were purposively selected for this study because the researchers had reasonable access to the target population.^6^

These public health clinics provide a wide range of primary health care services, which include immunisation, antenatal care, postnatal care, HIV and AIDS care, TB services, family planning, and care for acute and chronic conditions.

The target population were all professional nurses working in the public health clinics in Ekurhuleni for 2 years and more, who expressed willingness to share information with the researchers.^8,9,10^ Eight professional nurses were purposively sampled in order to select information-rich participants for the study and to best understand the central phenomenon.^8,9,10^ As the purpose of the study was to gain insight into the professional nurses’ experiences of caring for patients, participants were selected based on their first-hand experience with the phenomenon of interest. Purposive sampling was therefore used to provide a contextualised understanding of the participants’ experiences, which was not guided by a desire to generalise the research findings.^6^ The guiding principle in selecting the sample for this study was selecting cases that would most benefit the study. Yin^11^ indicates that all participants ought to have experienced the phenomenon and be able to articulate what it was like to have lived the experience. As one of the research objectives was to explore and describe the experiences of the participants, the researcher specifically looked for participants with diverse demographics who shared a common experience.

Prior to gaining entry to the research sites, the researchers had to negotiate with the authority of the health district for permission to enter into the clinics for study purposes. Before going into the field, the researchers identified the clinics that were to participate in the study.^6,11^ The researchers made arrangements with the managers of nursing services and facility managers in charge of the public health clinics to communicate their research endeavours with them. Recruitment was managed cooperatively with the intended participants by the facility managers, who assisted the researchers with identifying the relevant participants. The researchers controlled bias in identification of participants by the facility managers by ensuring that the facility managers knew what to look for in prospective participants.^11^ Once participants expressed a willingness to share information with the researcher, the participants were contacted individually, face to face, on the same day by the researcher to facilitate the establishment of rapport and trust with them. Interview dates and times were set by the researcher, agreed upon with participants on the day of the first meeting and were then communicated to the facility managers. On the day of the interview, facility managers and participants were contacted telephonically to remind them about the scheduled times for the interviews. Informed written consent and permission to voice-record the interview were obtained from the participants on the day of the interviews.^12^

### Data collection

Data were collected by the first author. In-depth, individual phenomenological interviews were conducted in English during working hours in quiet consulting rooms away from the patients’ waiting area. With permission from the participants, each interview was audiotaped. In-depth, individual phenomenological interviews were used to allow the researcher to gain insight and in-depth data from participants through exploring of their personal experiences. The interviews were unstructured as they were conversational and interactive in nature.^6^ The researcher did not have a set of prepared questions for the interviews but discussed the question of interest with the participants by asking them a single broad, open-ended question to describe their experiences of caring for patients in their clinics.^11^ Interviews lasted approximately 45–60 min, where participants were allowed to respond freely in order to provide the most information possible. During the interviews the researcher gave minimal responses as guided by the central question. Probes, prompts and summaries of participants’ last statements encouraged the participants to talk more about their experiences. The probes encouraged elaboration by participants, including questions such as ‘anything else?’, ‘and then what happened?’ as well as ‘tell me more about it’. Data collection continued until data saturation was reached.^6,8,9^ Data saturation was reached at the end of the fifth interview, when there was repetition of information. Three additional participants were recruited for verification and confirmation of the previously collected data. At the end of the eighth interview, the researcher was able to determine that the addition of new participants confirmed the findings rather than adding new information. The researcher did not predetermine the number of participants for the study beforehand but continued with data collection until data saturation was achieved. Observational and field notes were made as part of data collection. The researcher received training in conducting interviews. The researcher did not deviate from the role of being the data collection instrument but guided the interviews and maintained focus on the topic under investigation.^13^

### Data analysis

In the initial step of data analysis, the researcher transcribed the audio-recorded interviews verbatim. Transcription of the interviews was completed as soon as possible after the interviews. The transcribed interviews and field notes collected during the interviews were interpreted, which gave meaning to the data. Data analysis was conducted using Giorgi’s method, which is used in qualitative studies to identify the units of data, described as ‘the marking of what is of interest in the text’.^6,12^ Data were coded by developing and applying a list of codes to new segments of data when an appropriate segment was encountered. An independent coder, who was an expert with proven knowledge about qualitative research, also analysed the data to ensure the researcher’s objectivity and to reduce bias.^14^ The researcher and the independent coder had a consensus discussion about the findings, and these were recontextualised into the literature.

### Trustworthiness

Principles of trustworthiness according to Lincoln and Guba^15^ were adhered to.^6,9^ In-depth, individual interviews, field notes and observational notes were used to assure credibility. The researcher used well-established research methods and described them with sufficient detail so that the study can be replicated. Multiple literature sources such as articles, Internet searches and books were used to enhance credibility of the research findings. Bracketing was achieved by identifying and holding in abeyance preconceived beliefs and opinions about the phenomenon under study by the researcher. A reflexive diary was maintained by the researcher in an effort to bracket by making note of interests that would be taken for granted.^6,9^ Personal values were clarified; areas of bias and feelings that would indicate a lack of neutrality or possible conflict were identified. The researcher engaged in a social relationship with the participants, with the quality of the relationship individualised to every participant. Although the relationship needed to be friendly, it was not a friendship.^6,9^ Transferability was achieved by purposive sampling of participants and by a rich description of results with direct quotations from participants. Dependability was achieved by the code–recode method of analysis, where data were coded over an extended period of time to ensure consistency of the coding strategy.^6,9^ Furthermore, personal notes and field notes were kept. Confirmability was enhanced by a confirmability audit, where the study supervisors audited the research project. The researcher compared the identified codes with those of an independent coder. A consensus discussion was held between the researcher and the independent coder to discuss the findings.^6,9^

### Ethical considerations

Informed written consent was obtained from each participant before each interview commenced.^9,12^ With permission from the participants, each interview was audiotaped. Participants were informed that they could opt out of the research at any stage without being penalised.^9,12^ Numbers were used in the interview transcripts in adherence to the principle of anonymity and confidentiality. Research data and the master list of participants’ names and matching numbers were kept in a locked cupboard in the researcher’s office that could only be accessed by the researcher and the study supervisors. Information gathered about the participants was kept in a manner that did not connect them to the individual information.^9,12^ The principle of justice concerning fair treatment and the right to privacy and anonymity in maintaining the researcher–participant relationship was observed.^9,12^

Ethical approval to conduct the study was obtained before the study commenced from the Faculty of Health Sciences Research Ethics Committee (reference number REC-01-160-2016), Ekurhuleni District Ethics Committee (reference number GP-2016RP16-455), as well as the public health clinics (PHCs) where the study was conducted.

## Findings of the study

### Demographic profile of the participants

The eight participants in this study were female, and their ages ranged between 40 and 52 years. Five of the eight participants were in the age group 43–49 years while the remaining three were in the age group 50–52 years. All participants had a diploma in nursing and a diploma in primary health care nursing. Three of the eight participants had a bachelor’s degree in nursing. All participants had more than 2 years’ experience working in the public health clinics, of whom five had between 7 and 15 years’ work experience and the other three had between 27 and 32 years’ work experience. The age gap between the youngest and the oldest participant was 9 years. Seven out of the eight participants spoke 3 of the 11 official languages in South Africa, while one was Afrikaans speaking.

### Themes

Two themes emerged from the data, which were the experienced empowering and the experienced disempowering aspects of caring for patients. Table 1 presents the two themes and the categories that emerged from the data.

**TABLE 1:** Themes and categories.

Theme	Categories
Theme 1: Experienced empowering aspects of caring	1.1 Empowering interpersonal experiences Effective caring. Therapeutic use of self by professional nurses. Teamwork by professional nurses. Constructive management practices. Empowering experiences when affirmation is received from patients.
Theme 2: Experienced disempowering aspects of caring	2.1 Disempowering interpersonal experiences Negative attitudes of the professional nurses. Patients’ negative attitudes. Discrimination. Uncaring behaviours by the professional nurses. Absenteeism by the professional nurses. Language barriers. Perceived favouritism.
	2.2 Disempowering experiences with the public health clinic system Quantity versus quality: high workloads. Shortage of medicines, lack of functional medical equipment and other essential resources. Insufficient budget allocation. Shortage of professional nurses. Low salaries, no rewards and high resignations by professional nurses. Unavailable ambulances.

*Source*: Nesengani, TV. Strategies for professional nurses to facilitate caring of patients in the Primary Health Care clinics in Gauteng Province, South Africa [Unpublished D Cur thesis]. Johannesburg: University of Johannesburg; 2019.^38^

#### Theme 1: Experienced empowering aspects of caring

From the eight interviews conducted, it was discovered that the participants had empowering experiences that enabled them to render appropriate and acceptable care to their patients. Under this theme, the empowering experiences identified by participants include empowering interpersonal experiences and empowering experiences through client affirmation.

**Empowering interpersonal experiences:** Participants expressed empowering interpersonal experiences that seemed to enable them to render effective caring to patients. These empowering interpersonal experiences included effective caring and communication; therapeutic use of self by the professional nurses; teamwork by the professional nurses; constructive management practices and empowering experiences through client affirmation.

- **Effective caring and communication:** Participants expressed the value they attached to rendering effective caring to patients by doing the best they could while caring for patients. Participants revealed the following:
  ‘We are trying our best in caring for our patients. Also from my colleagues’ side, most of my colleagues are very positive towards our patients; they are caring and kind. In general, I feel that that our patients are well cared for. I will give them the best possible care that I can. (PN1 [Professional Nurse 1], female, 46 years old)
- **Therapeutic use of self by the professional nurses:** Participants narrated how they engaged themselves in therapeutic use of the self while caring for patients. This was expressed by participants in terms of how they maintained specific relationships with patients and how they accepted responsibility to effectively care for patients.
  ‘We will always be there, but we’ll be trying to make this [a] better and faster, and more effective system so that they can also reach their workplace faster. We are here to serve them, and we are here to help them. You must try and help them correctly because that’s what we are here to do.’ (PN7, female, 43 years old)
- **Teamwork by the professional nurses:** Teamwork was considered to be beneficial to participants and to their colleagues in enabling effective caring for patients. Participants expressed that teamwork enabled them and their colleagues to improve patient care planning in the workplace. Participants highlighted that teamwork was realised through helping each other as colleagues during times of need, which was facilitated through effective communication. Working as a team was instrumental in boosting the participants’ morale.

Some participants said:

‘Teamwork helps us to overcome such challenges such as shortage of staff. As I have been working in this clinic, some of the professional nurses try to create a more supportive working environment that facilitates sharing of expertise that enhances teamwork. When teamwork is there in the facility, we provide caring for our patients in a better way.’ (PN2, female, 49 years old)

- **Constructive management practices:** Participants indicated that having management that is involved and supportive was beneficial in creating an environment conducive to effectively caring for patients. Participants associated constructive management practices with appreciation shown by their managers for the effective caring for patients and with the motivation and the support managers gave them. Participants expressed the following:
  ‘The support that I have received in the past, I’m talking now from being a little bit of a junior nurse, stepping up to [become] a senior nurse, my support system was good. I had always had a very good support structure, from the senior nurses to managers. They taught me very well what the clinic is like, and what work we need to do in the clinic. They will teach you the correct thing; they are more than willing to support.’ (PN7, female, 43 years old)

**Empowering experiences through client affirmation:** Participants revealed the good feelings they had when patients appreciated the effective caring they received while utilising the public health clinics. Patients’ appreciative behaviours were perceived by participants as empowering and boosting their morale. This was revealed by participants:

‘Most of our patients appreciate the care that we provide to them. Most of our patients are appreciative of our services, especially the elderly. Even in the suggestion box, we get a lot of compliments to say they are happy with our service. Others do phone our manager to compliment the staff. During Christmas time, others do bake cakes and bring them for us in the clinic. So, in that manner, I can say our patients are happy.’ (PN1, female, 46 years old)

#### Theme 2: Experienced disempowering aspects of caring

From the interviews conducted, it became clear that participants also had disempowering experiences that affected their caring for patients. Categories identified under this theme were disempowering interpersonal experiences and disempowered caring because of public health clinic system challenges.

**Disempowering interpersonal experiences:** Disempowering interpersonal aspects of caring seemed to negatively affect and disenable the participants from rendering effective caring for patients. Participants with disempowering interpersonal experiences are more likely to be demotivated in the workplace. These disempowering interpersonal experiences included negative attitudes of the professional nurses; patients’ negative attitudes; discrimination by the professional nurses; uncaring behaviours by the professional nurses; absenteeism by the professional nurses; language barriers and perceived favouritism.

**Negative attitudes of the professional nurses:** Some participants expressed that they noticed their colleagues displaying negative attitudes to patients. Negative or poor attitudes led some professional nurses to become inconsiderate and impatient while caring for patients:

‘This also goes with the type of attitude towards the patient and the condition. Our attitudes count a lot when it comes to caring for patients, especially the foreign patients, though our local patients are met with such attitudes as well. Immediately you have such type of an attitude, you are not going to render proper care to patients.’ (PN1, female, 46 years old)

**Patients’ negative attitudes:** Some participants revealed that patients are very much unappreciative of the care they receive from the professional nurses. The participants’ perception of patients’ negative attitudes appeared to have strongly influenced their nurse–patient relationships.

‘Patients are very much unappreciative. When I gave them their monthly supply, they would go to the other sisters and try and check if I gave them the correct treatment. Others were still doubtful about my capabilities; this type of attitude really upset me.’ (PN1, female, 46 years old)

**Discrimination by the professional nurses:** Participants expressed having experienced acts of discrimination in the public health clinics, which were described as acts of failing to accept new colleagues by those who had been long in the service. Some participants revealed that discrimination was also directed towards patients living with HIV as well as patients who were foreign nationals. Discrimination directed towards patients who were foreign nationals led the professional nurses to treat these patients differently from the rest of the patients:

‘Remember, this HIV condition still has stigma, where even us as professionals, we still discriminate against patients with such diagnoses. I have seen nurses who are still discriminating against such patients. Patients are still suffering in the hands of professionals, where they are also called names.’ (PN1, female, 46 years old)

**Uncaring behaviours by the professional nurses:** Patients who experienced uncaring behaviours by the professional nurses were observed to be treated in a rude manner. Some participants revealed that they did not show concern nor empathy for the patients while caring for them:

‘I don’t actually feel any emotions for these patients as we are supposed to spend less time with them. I just work and try to get the service running. Many nurses don’t care anymore; many are just working for a salary without showing any compassion to the patients.’ (PN3, female, 52 years old)

**Absenteeism by the professional nurses:** Absenteeism by the professional nurses was perceived by participants to have a negative impact on the morale of the remaining staff. The high absenteeism rate among the professional nurses influenced the other colleagues to stay away from work as well, which was regarded as disempowering from effective caring for patients:

‘When I arrive at the clinic and realise that other nurses are not on duty, I also feel like going back as well because I know, those who are on duty, we won’t cope with the number of patients that day. What I have learned these days is to absent myself as well because I become overworked. Other nurses can be absent for a week and come back with a medical certificate, so I also do it these days.’ (PN3, female, 52 years old)

**Language barriers:** Language barriers were considered disempowering by participants as they had to struggle in communicating with patients, especially those patients who were foreign nationals. Participants revealed that language barriers prevented them from acting upon some patients’ needs because of the inability to effectively communicate with those patients:

‘Communication with such patients is not that effective; cards are written in those foreign languages that I would not understand – therefore, I also struggle getting the proper history from the patient. We struggle a lot to do the right thing because of the language barriers.’ (PN3, female, 52 years old)

**Perceived favouritism:** Participants accused their managers of giving preferential treatment to some of their colleagues. Perceived favouritism by managers was revealed to have caused dissatisfaction among some participants:

‘One of the worst experiences is that preferences in incentives such as half days was given to staff members who have been working long in the facility than the rest of the other nurses. This made me feel bored and angry because most of the time, those nurses would even leave some of the patients from their queues.’ (PN2, female, 49 years old)

### Disempowered caring because of public health clinic system challenges

Disempowering experiences with the public health clinic system seemed to disenable the participants from rendering effective caring for patients. Part of caring is providing a wide range of primary health care services, which include immunisation, antenatal services, HIV and AIDS care, TB services, family planning and care for acute and chronic conditions. Participants indicated that they were discouraged and demotivated as they usually had to ask for resources from other clinics before they started working. The disempowering experiences with the clinic system included high workloads; shortage of medicines, lack of functional medical equipment and other essential resources; insufficient budget allocation; shortage of professional nurses; low salaries, no rewards and high resignations by professional nurses; and unavailable ambulances.

#### Quantity versus quality: High workloads

The participants voiced that their employer was only concerned with the number of patients they had to see on a daily basis. Participants compared the high number of patients to quantity, instead of the quality of care they were supposed to render to patients. High workloads compelled the participants to work under pressure:

‘What we are doing is quantity instead of quality. You always struggle with seeing all patients in a day. We actually become overwhelmed with the number of patients that we are supposed to see on a daily basis. I would do all the slap dashes in order to finish the queue allocated to me, which I consider as not appropriate caring for the patients.’ (PN1, female, 46 years old)

#### Shortage of medicines, lack of functional medical equipment and other essential resources

Shortage of medicines, lack of functional medical equipment and other essential resources in the clinics were some of the disempowering experiences raised by participants. Participants revealed that they usually had to ask for resources from other clinics in order to ensure that patients receive effective caring:

‘There are shortages of resources; you have to ask for the resources from other clinics. We don’t have resources; you have to ask for resources from other clinics, which further hampers caring. Another thing, the issue of shortage of medication still makes caring a problem. We struggle with the shortage of various medications.’ (PN1, female, 46 years old)

#### Insufficient budget allocation

Some participants revealed how insufficient budget allocation for their clinics contributed to their disempowering experiences. The inability to purchase medical equipment and other essential resources was perceived by the participants to be the result of insufficient budget allocation for the clinics. Shortage of functional medical equipment and other essential resources was expressed by participants to be making effective caring for patients impossible:

‘Few resources are purchased, and the reason for the shortage of vital resources is that there is not enough budget. All we hear when we raise the issue of shortage of vital resources is that there is not enough budget; we have to wait for the next financial year. When the new financial year starts, still, few resources are purchased.’ (PN2, female, 49 years old)

#### Shortage of professional nurses

Shortage of nursing staff in the clinics was perceived as disempowering by the participants, as they were expected to do more in terms of their duties. Participants revealed that despite the shortage of nursing staff, all the healthcare services in the clinics had to run daily, at the same time, by the available staff:

‘Some of the challenges that we go through is the human resource problem: we don’t have enough nurses. I feel that some of the times we don’t have enough nurses to cater for the specialised services. If I can give you an example, when it comes to antenatal care, there are a lot of things to be done. There are papers that need to be completed, so I feel that one nurse alone tackling that department is a bit overwhelming.’ (PN1, female, 46 years old)

#### Low salaries, no rewards and high resignations by professional nurses

Low salaries and lack of rewards were perceived by participants to be the driving forces behind the high number of resignations by professional nurses. Unfavourable work conditions appeared to strongly influence the professional nurses in a negative way, leading them to be demotivated and to resign in high numbers:

‘I am not rewarded; maybe at the end of the day, a better salary. The department is not paying people; I’m looking at things now like salary increases. Then obviously and unfortunately I will have to look for another job opportunity where I will get more job satisfaction, and maybe at the end of the day, a better salary.’ (PN7, female, 43 years old)

#### Unavailable ambulances

The unavailability and long turnaround time of the ambulances to transfer patients to hospital was perceived to be a risk to the patients’ lives by the participants. Participants revealed that sometimes when they needed an ambulance to transfer patients to hospital, the ambulance never came:

‘The other thing is the issue of the ambulance. Most of the time when we have patients who need to be transferred to the hospital, we usually find it being a challenge because you can phone an ambulance, [but] the ambulance will take three to four hours before it comes; sometimes no ambulance will come to the clinic to fetch the patient.’ (PN4, female, 49 years old)

## Discussion

Based on the findings of this study, it is conclusive that participants had empowering and disempowering experiences of caring for patients. Participants had a sense of value in the empowering experiences, which were perceived as enablers in rendering effective caring for patients; while the disempowering experiences were perceived to have hindered them in effective caring for patients. The empowering experiences of caring identified by some participants were effective caring and communication, while others had disempowering experiences identified as uncaring behaviours by professional nurses. Overall, nursing is viewed as a caring practice whose science is guided by the moral art and ethics of caring and responsibility.^16,17^ Effective caring is frequently referred to as the moral human trait, ethic or ideal at the heart of nursing, while simultaneously being viewed as an ideal service to the weak and vulnerable.^18,19^

The nursing profession requires nurses to show effective caring behaviours that include comfort, compassion, concern and empathy. Participants indicated that empowering interpersonal experiences were more helpful in motivating them to effectively care for patients.^20^ Empowering interpersonal experiences were essential for participants to work together, discuss various issues and reach solutions that benefited both the employees and the organisation. Some participants voiced the issue of uncaring behaviours displayed by colleagues towards patients, which they described as disempowering in effective caring for patients. Issues relating to uncaring behaviours stand in stark contrast to the popular stereotype of the caring, selfless, healing profession with a vocation epitomised by Nightingale and her peers. Despite all of this, Florence Nightingale remains the touchstone for compassion in the nursing profession. Explanations on the concerns about caring from various countries have however been drawn in the attempt to shed light on this darker side of caring, where neglect, incompetence and occasionally abuse have been evident. There are indicators that care failures have occurred as a result of an absence of compassion among care deliverers, while situational and contextual factors might also be better reference points in the journey to understanding. The broad argument points brought forward were the possibility that the issues were a result of social organisational factors and a lack of compassion on the part of the healthcare staff.^21^

Therapeutic use of self was identified as an empowering experience while caring for patients by the participants. Therapeutic use of self was related to caring as a therapeutic intervention, an interpersonal interaction, a moral imperative, an effect and a human trait. Therapeutic use of self-enabled the participants in this study to show compassion and willingness to work towards the realisation and satisfaction of the patients’ health needs. Their act was shaped by a relationship between the nurse, the patient and the moral values of the nursing profession. The nursing profession is characterised by high moral standards, including commitment to the well-being of others. These continue to be prerequisites in maintaining and strengthening the patients’ sense of dignity. Compassion and a willingness to help are considered vital assets in the nursing profession that not only allow nurses to establish a therapeutic relationship with patients but also to provide high-quality nursing care.^22^ According to a study conducted on primary care providers’ experiences of caring for complex patients in primary care by Loeb et al.,^23^ participants revealed that developing a trusting relationship with their patients was therapeutic for the patients. This was regarded as fundamental to their ability to provide optimal care.

Other empowering interpersonal experiences such as teamwork by the professional nurses were perceived as beneficial for effectively caring for patients. Teamwork is a core competency for nursing practice and enables nurses to function effectively as members of teams. When everyone works together, it will help prevent errors from occurring and help nurses to reach their goal of providing optimal nursing care.^1^

Some participants indicated that the role played by their managers was satisfying as they engaged themselves in constructive management practices that contributed towards effective caring for patients, while others perceived favouritism. Favouritism is defined as the act of unfairly treating some people better than others because one likes them better.^24^ Favouritism is described as a major reason managers fail to gain their employees’ respect. When favouritism prevails in the workplace, some very good employees typically quit or leave the department.^25,26^ Participants who perceived favouritism expressed dissatisfaction because of managers who showed favouritism to some colleagues in the public health clinics without real merit. Such employees were subject to special treatment while the newly employed professional nurses got pushed aside. The favoured employees incessantly got rewards in the form of half days awarded by the managers. Literature reveals that staff members who feel that their manager sincerely supports and cares about them and the work they do are enabled to pass that feeling of caring on to their patients and other customers. Nurses working in supportive work environments are reported to be more satisfied in their jobs, experience less burnout and report better nursing care quality.^27,28^

In addition to the empowering interpersonal experiences, there were empowering experiences when professional nurses received affirmation from patients. Participants expressed a good feeling brought about by patients who appreciated the caring they received while utilising the public health clinic system. Patient satisfaction is regarded as a significant factor leading to patient loyalty and increased staff morale. Increased personal and professional satisfaction was felt by participants who revealed feeling happy about the positive feedback they received from patients.^29,30^ According to a study conducted by Takazori et al.^31^ about primary care providers’ experiences of caring for complex patients, it was revealed that participants experienced a personal reward in getting to know their patients well.

Some participants indicated that they experienced negative attitudes from their colleagues and from patients. Participants associated negative attitudes displayed by colleagues with being overworked, which had a negative influence on effective caring for patients. Findings regarding negative attitudes by professional nurses in public health clinics have been substantiated in previous research. Research confirms that South African hospitals and clinics are reported to experience these negative incidents.^32^ Previous studies illustrate ‘attitude’ in a person’s tone of voice but which is often communicated loudly and clearly through non-verbal communication. Considering a negative or a poor attitude conjures up a picture of a person who looks miserable. The person gives hostile or disinterested verbal responses, and exhibits body language suggesting he or she would rather be somewhere else.^33,34^ Often the attitude that accompanies a verbal interaction, which can be positive or negative, is described as being much more meaningful than the actual words spoken. Working in the health care context requires special skills and an attitude that supports services to others.^34,35^ Takazori et al.^31^ further indicate that in their study on nurses’ experiences of caring for patients with HIV and AIDS in Ardabil, Iran, it was revealed that some participants treated patients with disrespect, with disregarding behaviours being displayed towards patients with HIV or AIDS.

Some participants raised the issue of being discriminated against by their colleagues while they were newly employed in the public health clinics, which is also regarded as bullying. Bullying is described as behaviours that include verbal abuse, threats, continual criticism, demeaning remarks, intimidation and undermining, as well as more subtle behaviours such as refusing to cooperate, being unavailable to give assistance, hampering another’s performance and making their work difficult.^21^ Other aspects of discrimination in this study were illustrated by the acts of some professional nurses, who discriminated against patients who were foreign nationals. The success of the acceptance of other human beings is determined by the empathetic understanding of each person, which enables people to build a trusting relationship, based on respect.^21^

Participants in this study also revealed they had resorted to absenteeism, which was a result of high workloads. ‘Absenteeism’ is defined as being frequently away from work or school, especially without good reasons.^35^ Absenteeism is also described as a habitual pattern of absence from a duty or obligation. Literature indicates that workers who report being overloaded with work tend to avoid stress by withdrawing from the workplace.^36^

Language barriers were also regarded as a disempowering experience by participants. Participants revealed that some patients could not express themselves in the languages that they as professional nurses knew, which they indicated hindered communication and caring in a true sense. Language barriers are defined as difficulties experienced by people speaking different languages.^24^ It has been confirmed that despite the attention of nurses and patients to effective communication, there are some barriers.^30^

Participants revealed feeling disempowered because of the public health care system challenges, which they expressed as various aspects that affected rendering of effective caring for patients. Within the context of the important contributions that professional nurses make in patients’ lives, many are found to be challenged to render effective caring because of barriers. The barriers expressed by participants in this study included high workloads; shortage of medicines, lack of functional medical equipment and other essential resources; insufficient budget allocation; shortage of professional nurses; low salaries, no rewards and high resignations by the professional nurses; as well as unavailable ambulances. Participants revealed that they had to see a high number of patients on a daily basis. High workloads are associated with the volume of nurses’ work, which is commonly measured by staffing levels or nurse–patient ratios. When nurses experience heavy workloads, they tend to leave essential tasks undone, resulting in negative nurse and patient outcomes, with the quality and safety of patient care being compromised. Heavy workloads interfere with the nurses’ ability to live out care according to a caring philosophy. Heavy workloads put some nurses under pressure to arrive early or leave late, missing breaks because they do not have enough time to get their work done, while they have too much to do. The main work-related reason given by nurses when they leave their jobs is usually that of working under pressure. Nurses who encounter heavy workloads in their workplace end up experiencing burnout or emotional exhaustion, which has been linked to higher rates of absenteeism.^22,28^ Loeb et al.^23^ further indicate in their study that participants expressed frustration over productivity demands, where there was no ability to have more time with individual patients.

Shortage of medicines kept participants from prescribing relevant medicines for the patients. The Treatment Action Campaign and SECTION27 reveal that there has been a sharp deterioration in health care at hospitals and clinics in the Gauteng Province over the past few years, marked by shortages of medicines, collapsing infrastructure, broken equipment, inadequate provision of staff, misuse and misallocation of funds.^37^ Insufficient budget for new equipment has been noted to cause delays and difficulties in getting equipment ordered. Participants in this study expressed that lack of functional medical equipment caused them to fail in rendering effective caring for patients.

Shortage of professional nurses in the public health clinics made it difficult for the participants to provide effective caring for patients. Staffing is described as ensuring that an adequate number and mix of the health care team members are available to provide safe, quality patient care. Nurses working in the clinics that are short-staffed are likely to experience high levels of emotional labour, which leads to poor psychological and physical health, increased sick leave and poor staff retention.^28^ Participants in this study revealed that the high resignations by professional nurses in the public health clinics were associated with a lack of personal motivation. Staff shortages in most instances are attributed to low pay, which is significant in staff retention problems, particularly among nursing personnel.^25^ The outcome of these developments puts nursing under pressure, because nursing takes time to perform. Essential care is then left undone while a shortage of professional nurses leads them to experience moral distress.^25^

Participants revealed the experience of unavailability of ambulances to transfer patients to hospitals as risky and having a negative impact on effective caring for patients. Reports indicate that while the Gauteng Province is not the only province affected by a lack of ambulances, the crisis in emergency medical services and patient transport is acute in the Eastern Cape as well. This extends to other provinces, with the Free State and Mpumalanga perhaps chief amongst them.^37^

### Strengths and limitations of the study

The strength of this study relates to the methodology used, where in-depth, individual interviews were used to collect data. The use of purposive sampling enabled the researcher to select information-rich subjects and allowed participants to voice their views and experiences related to the study topic. A limitation for this study was that a small number of participants were interviewed; however, purposive sampling was used to provide a breadth of understanding from the participants. This will aid in the potential transferability of findings to similar health care practitioners. Another limitation was that study participants were all between the ages of 40 and 52 years and were female. This was because of the lack of male professional nurses and the unavailability of candidates who matched the inclusion criteria with regard to age in the public health clinics where the study was conducted.

### Recommendations

To facilitate effective caring for patients in Ekurhuleni, it will be beneficial to nurture and maintain the empowering aspects of caring among professional nurses in order to enhance job satisfaction and effective caring for patients. To facilitate addressing professional nurses’ disempowering experiences in the public health clinics in Ekurhuleni, it is of crucial importance to address the professional nurses’ negative attitudes, discrimination, uncaring behaviours and absenteeism through adherence to relevant policies. It will also be beneficial to institute measures such as having monthly meetings between management and the professional nurses in order to discuss issues relating to effective caring for patients. Further research needs to be conducted in the public health clinics to explore patients’ experiences of caring.

## Conclusion

This study identified that participants had empowering and disempowering experiences while caring for patients in the public health clinics in Ekurhuleni. The experienced empowering aspects of caring enabled participants to render effective caring for patients, while the experienced disempowering aspects of caring were perceived by participants as negatively affecting them in rendering effective caring for patients.

## References

[1] Peng X, Liu Y, Zeng Q Caring behavior perceptions from nurses of their first-line managers. Scand J Caring Sci. 2015;29(4):708–715. 10.1111/scs.12201 25648604

[2] Rhodes KM, Morris AH, Lazenby RB Nursing at its best: Competent and caring. Online J Issues Nurs. 2011;16(2):10 10.3912/OJINVOL16NO02PP701 22088159

[3] Begum S, Slavin H Perceptions of caring in nursing education by Pakistan Nursing Students: An exploratory study. Nurse Educ Today. 2012;32(3):332–336. 10.1016/j.nedt.2011.10.011.2011 22071274

[4] Department of Health Consolidated report on inspections of primary health care delivery sites. Pretoria: The Public Service Commission; 2010.

[5] Babbie E, Mouton J The practice of social research 10th ed. Cape Town, Republic of South Africa: Oxford University Press Southern Africa; 2017.

[6] Polit DF, Beck CT Nursing research: Generating and assessing evidence for nursing practice 10th ed. Philadelphia, PA: Wolters Kluwer; 2017.

[7] Ekurhuleni Metropolitan Municipality Development Guide [homepage on the Internet] 2010 [cited 2018 Aug 28]. Available from: http://www.ekurhuleni.gov.za

[8] Plano Clark VL, Creswell JW Understanding research: A consumer’s guide Boston, MA: Pearson Education; 2010.

[9] Holloway I, Wheeler S Qualitative research in nursing and healthcare 3rd ed. West Sussex: John Wiley; 2010.

[10] Moule P, Goodman M Nursing research: An introduction 2nd ed. London: Sage; 2014.

[11] Yin RK Qualitative research from start to finish 2nd ed. London: The Guilford Press; 2016.

[12] Dhai A, McQuoid-Mason D Bioethics, human rights and health law: Principles and practice Cape Town: Juta; 2011.

[13] De Vos AS, Strydom H, Fouché CB, Delport CSL Research at grass roots: For the social sciences and human service professions 4th ed. Pretoria: Van Schaik Publishers; 2014.

[14] Green J, Thorogood N Qualitative methods for health research London: Sage; 2014.

[15] Lincoln YS, Guba EG Naturalistic inquiry Newbury Park, CA: Sage; 1985.

[16] Mellish JM, Oosthuizen AM, Paton F An introduction to the ethos of nursing Johannesburg: Heinemann; 2010.

[17] Dahlke S, Wall S Does the emphasis on caring within nursing contribute to nurses’ silence about practice issues? Nurs Philos. 2017;18(3):1 10.1111/nup.12150 27699966

[18] Delves-Yates C, editor Essentials of nursing practice. London: Sage; 2015.

[19] Hawke-Eder S. Can caring be taught? Kai Tiaki Nurs New Zealand. 2017;23(3):23–46. 30549573

[20] Armstrong N, Hebert G, Aveling EL, Dixon-Woods M, Martin G Optimizing patient involvement in quality improvement. Health Expect. 2013;16:e36–e47. 10.1111/hex.12039 23374430 PMC3883095

[21] Stenhouse R, Ion R, Roxburgh M, Devitt PF, Smith SDM Exploring the compassion deficit debate. Nurse Educ Today 2016;39:12–15. 10.1016/j.nedt.2016.01.019 27006028

[22] Anderson EK, Willman A, Sjöström-Strand A, Borglin G Registered nurses views of caring in coronary care – A deductive and inductive content analysis. J Clin Nurs. 2015;24(23–24):3481–3493. 10.1111/jocn.12975 26335244

[23] Loeb DF, Bayliss EA, Candrian C, De Gruy FV, Binswanger IA Primary care providers’ experiences of caring for complex patients in primary care: A qualitative study. BMC Fam Pract. 2016;17:34 10.1186/5/287-016-0433-2 27004838 PMC4804627

[24] Hornby AS Oxford advanced learner’s dictionary 9th ed. Oxford: Oxford University Press; 2015.

[25] Cherry B, Jacob S Contemporary nursing: Issues, trends and management 6th ed. St. Lewis: Elsevier and Mosby; 2014. ISBN: 9780323101097.

[26] Koy V, Yunibhand J, Angsuroch Y, Fisher ME Relationship between nursing care quality, nurse staffing, nurse job satisfaction, nurse practice environment and burnout: Literature review. Int J Res Med Sci. 2015;3(8):1825–1831. 10.18203/2320-1612.ijrms20150288

[27] Büte M The effects of nepotism and favoritism on employee behaviors and human resources practices: A research on Turkish public banks. TODAiE’s Rev Public Admin. 2011;5(1):185–208.

[28] MacPhee M, Dahinten VS, Havaei F The impact of heavy perceived nurse workloads on patients and nurse outcomes. Admin Sci [serial online]. 2017 [cited 2018 May 31];7(1). Available from: www.mdpi.com/journal/admsci

[29] Lee TH Why patient loyalty matters and how to enhance it. Fulfilling patients’ needs. Health Care Financial Management [homepage on the Internet]. 2014 [cited 2018 May 31]. Available from: www.hhnmag.com 25647931

[30] Prakash B Patient satisfaction. J Cutan Aesthetic Surg. 2010;3(3):151–155. 10.4103/0974-2077.74491 PMC3047732 21430827

[31] Takazori Z, Moshfeghi SH, Karimollahi M Nurses’ experiences of caring for patients with HIV/AIDS in Ardabil, Iran. HIV Curr Res. 2017;2:118.

[32] Haskins JLM, Phakathi S, Grant M, Horwood CM Attitudes of nurses towards patient care at a rural district hospital in the KwaZulu-Natal province of South Africa. Afr J Nurs Midwifery. 2014;16(1):32–44. 10.25159/2520-5293/1485

[33] Sallee J Rational inattention and energy efficiency. J Law Econ. 2014;57(3):781–820. 10.1086/676964

[34] Doane HG, Varcoe C How to nurse? Relational inquiry with individuals and families in changing health and health contexts Philadelphia, PA: Lippincott, Williams & Wilkins; 2015.

[35] Molefe J, Sehularo LA Nurses’ perceptions on factors contributing to job dissatisfaction in a public psychiatric hospital in North West Province, South Africa. Afr J Phys Health Educ Recreat Dance. 2015;21(2):472–482.

[36] Norouzinia R, Aghabarari M, Shiri M, Karimi M, Samami E Communication barriers perceived by nurses and patients. Global J Health Sci. 2016;8(6):65–74. 10.5539/qjhs.v8n6p65 PMC4954910 26755475

[37] Treatment Action Campaign + Section 27 Providing quality healthcare for all [homepage on the Internet]. 2015 [cited 2018 May 31]. Available from: https://tac.org.za/campaigns/treatment-for-all/

[38] Nesengani TV Strategies for professional nurses to facilitate caring of patients in the Primary Health Care clinics in Gauteng Province, South Africa [Unpublished D Cur thesis] Johannesburg: University of Johannesburg; 2019.

